# An online clinical decision tool to screen for vertebral fragility fractures (Vfrac) in older women presenting with back pain in general practice: protocol for a feasibility study in preparation for a future cluster randomised controlled trial

**DOI:** 10.1007/s11657-023-01364-1

**Published:** 2024-02-07

**Authors:** Tanzeela Y. Khalid, Tim J. Peters, Lucy V. Pocock, Sarah Drew, Sarah A. Hardcastle, Zoe Paskins, Sarah Davis, Eleni P. Kariki, Emma M. Clark

**Affiliations:** 1 Musculoskeletal Research Unit, Translational Health Sciences, Learning and Research Building, Southmead Hospital, Bristol Medical School, University of Bristol, Bristol, BS10 5NB UK; 2 Bristol Dental School, University of Bristol, Bristol, UK; 3 Population Health Sciences, Bristol Medical School, University of Bristol, Bristol, UK; 4 Rheumatology, Royal National Hospital for Rheumatic Diseases, Bath, UK; 5 School of Medicine, Keele University, Staffordshire, UK; 6 School of Health and Related Research, University of Sheffield, Sheffield, UK; 7 School of Allied Health Professions, Faculty of Medicine and Health Sciences, Keele University, Keele, UK

**Keywords:** Vertebral fractures, Back pain, Osteoporosis, Vfrac

## Abstract

**Summary:**

This feasibility study for a future definitive randomized trial assesses the use and acceptability of a new clinical decision tool to identify risk of a vertebral fracture and those who should be referred for spinal radiography in women aged 65 or over presenting to primary care with back pain.

**Purpose:**

Approximately 12% of older adults have vertebral fragility fractures, but currently fewer than one-third are diagnosed, potentially limiting access to bone protection treatment. Vfrac is a vertebral fracture screening tool which classifies individuals into high or low risk of having a vertebral fracture, allowing targeting of spinal radiographs to high-risk individuals. The objective of this study was to investigate the feasibility of conducting a cluster randomized controlled trial to evaluate the use of an online version of Vfrac in primary care.

**Methods:**

The study will run in six general practices, with three given the Vfrac tool for use on older women (> 65 years) consulting with back pain and three using standard clinical processes for managing such back pain. Anonymised data covering a 12-month period will be collected from all sites on consultations by older women with back pain. Focus groups will be undertaken with healthcare professionals and patients on whom the tool was used to understand the acceptability of Vfrac and identify factors that impact its use. These patients will be sent a paper version of the Vfrac questionnaire to self-complete at home. Outputs of the self-completion Vfrac (high versus low risk) will be compared with the face-to-face Vfrac (high versus low risk), and agreement assessed using Cohen’s kappa.

**Results:**

This study will evaluate the use and acceptability of Vfrac within primary care and determine if data on resource use can be collected accurately and comprehensively.

**Conclusions:**

This article describes the protocol of the Vfrac feasibility study.

**Trial registration:**

ISRCTN18000119 (registered 01/03/2022) and ISRCTN12150779 (registered 10/01/2022).

## Background

There are approximately 3.5 million people in the UK living with osteoporosis [1] and more women are affected with this condition than men, with 1 in 2 women over the age of 50 breaking a bone because of it [2]. Vertebral fragility fractures (VFFs) are of particular importance, as they identify people at a high risk of future fractures, which can lead to morbidity, disability and reduced health-related quality of life [3, 4]. Treatment with bone protection therapies are available to reduce the risk of further fractures by 30–70% [5, 6]. The problem is that many patients fail to receive a diagnosis of VFFs, with estimates suggesting that over two-thirds remain undiagnosed [7]. There are many reasons for this important healthcare gap, including lack of clinical signs specific to vertebral fracture, the high prevalence of all-cause back pain in older people, and difficulty in understanding who should have spinal X-rays.

To address this, we have developed the Vfrac clinical decision tool to help primary healthcare practitioners decide if an older woman with back pain is at high risk of a VFF and therefore requires a spinal radiograph to confirm the diagnosis. Vfrac is targeted for use during consultations between a healthcare professional and an older woman consulting with back pain. Vfrac is the only evidence-based decision tool developed from research involving women with and without VFFs including qualitative[8], cross-sectional [9–11] and case–control studies [12]. The Vfrac tool comprises 15 questions based on self-reported data and a physical examination and takes approximately 5 minutes to perform. It has been made available on a web-based online interface to make it accessible for use across different IT systems in primary care in the UK. Use of the pre-determined cutoff [13] produces the output (“low risk—spinal X-ray is not recommended”—or “high risk—spinal X-ray is recommended as may have a vertebral fracture”). As previously published [14], the Vfrac clinical tool has an area under the curve (AUC) of 0.802 (95% CI 0.764–0.840). Statistical modelling shows no evidence of over/underfitting; optimism in the estimate of the AUC was 0.019 estimated using 500 bootstrapped samples; and multiple imputation to account for the missing data produced results that were similar to our final model and provides internal validation. A pre-trial economic evaluation, also published [14], calculates the incremental cost-effectiveness ratio (ICER) for Vfrac compared to standard care is £17,000 per QALY and Vfrac has the potential to be cost-effective but a definitive trial is warranted.

Prior to this study, no testing of Vfrac has been undertaken within a real-world clinical setting and this is necessary to understand if and how it will be used, and whether changes to the tool are required to optimise its use. Primary care service delivery has changed in the face of the COVID-19 pandemic, and it is unlikely that there will be a full-scale return to face-to-face consultations with GPs as the primary mode of clinical assessment. This warrants an investigation as to whether the Vfrac tool can be used remotely by patients, and this is the first aim of this feasibility study. Specifically, to assess whether patients can self-complete the questionnaire at home and whether the results will be in agreement with the use of the Vfrac tool by healthcare professionals in primary care. The second aim of this study is to investigate the acceptability of Vfrac as an online clinical decision tool in primary care, as this will help identify barriers and facilitators to its implementation and delivery for the design of a future definitive trial of Vfrac.

## Methods

### Feasibility study design

**Fig. 1:**
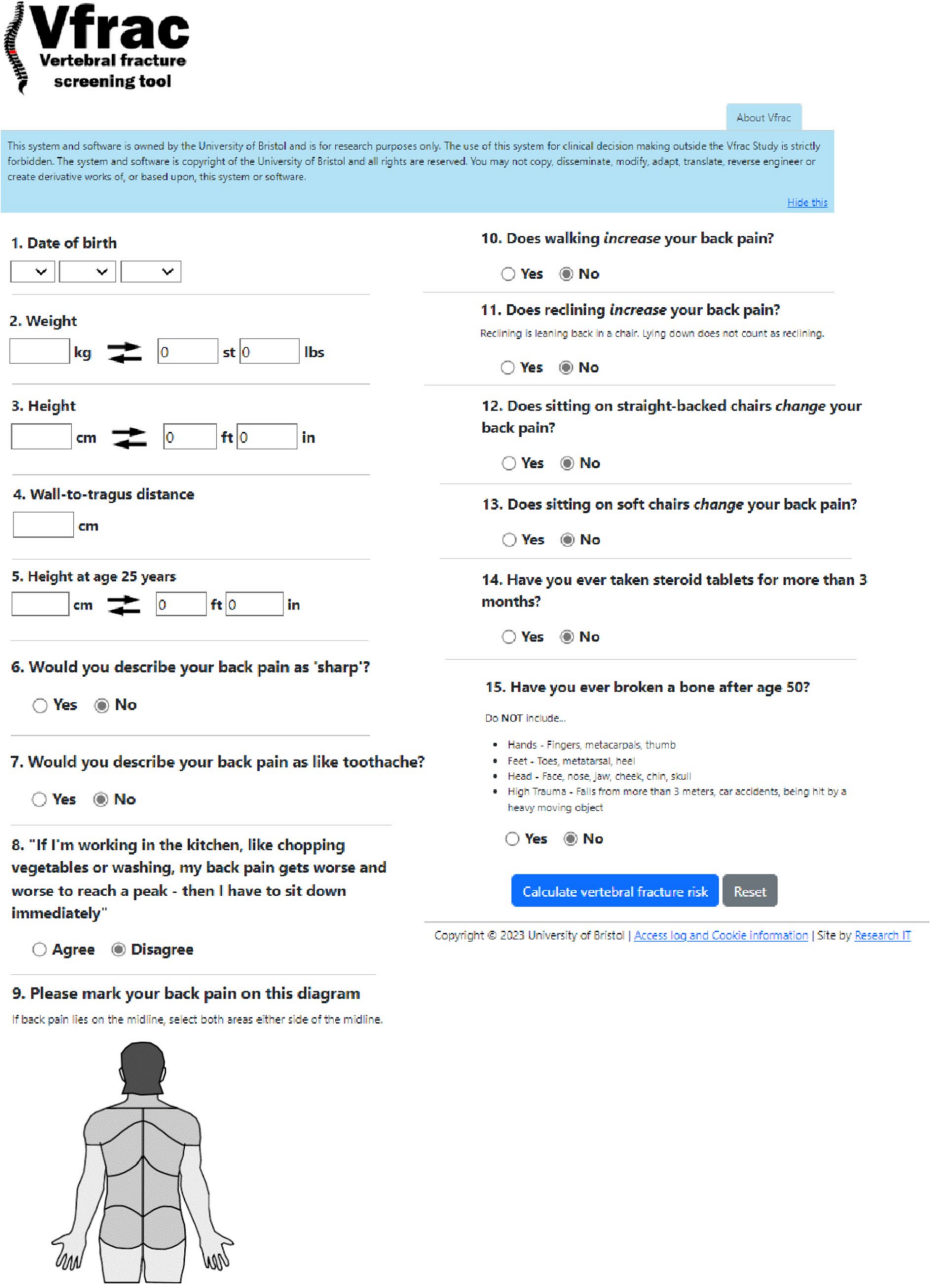
The online vertebral fracture screening tool made available for use by general practitioners in three general practices across the West of England Clinical Research Network

**Fig. 2:**
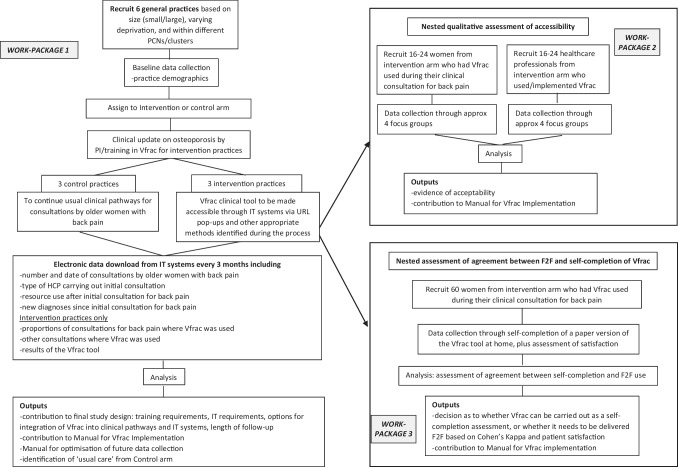
Overview of study processes for each of the three work packages of the Vfrac feasibility study

A screenshot of the online Vfrac tool is given in Fig. 1. Vfrac is a multicentre feasibility study, with nested evaluations. There are three work packages (WP), the objectives of which are summarised in Table 1. An overview of the study processes in each of the work packages is provided in Fig. 2**.**

**Table 1:** Work packages of the Vfrac feasibility study

Work Package (WP)	Vfrac study component	Objective(s)
1	Vfrac implementation within primary care	To optimise integration of the Vfrac decision tool within primary care IT systems and clinical pathways. To determine the length of follow-up (from assessment to final management) that is required for a future trial.
2	Nested qualitative assessment of acceptability of the Vfrac tool	To understand how acceptable Vfrac is to healthcare professionals (providers) and patients (recipients) and identify factors that impact on implementation, including barriers and facilitators to delivery
3	Nested assessment of agreement between the use of Vfrac within general practices and self-completion at home	To decide whether Vfrac can be self-completed at home or whether it needs to be delivered face-to-face for any future trial.

### Regulatory approvals

Ethics approval was obtained from Yorkshire & The Humber—Bradford Leeds Research Ethics Committee on 28th June 2022 (reference 22/YH/0135) and Health Research Authority approval on 28th June 2022. The study is registered on the International Standard Randomised Controlled Trial Number registry (the ISRCTN reference covering work packages 1 and 2 is ISRCTN18000119 and work package 3 is covered by the ISRCTN reference ISRCTN12150779).

### Patient and public involvement (PPI) in study design and delivery

The design of this feasibility study has been actively discussed with our Patient Experience Partnership in Research group (PEP-R), to seek their views and thoughts, and to modify our processes and strategies as necessary. To date, seven members of the PEP-R group have contributed to the study design—in particular, our recruitment strategies, conduct of the patient interviews and focus groups, the development of all patient-facing materials to improve wording and readability, and some administrative changes. PPI in our governance structure is also key; two people with osteoporotic vertebral fractures have agreed to take an active part in our Steering Committee, which will meet every 6 months.

## WP 1: Vfrac implementation within primary care

For this work package general practices will be recruited and then assigned to either intervention or control by purposeful selection to ensure there is variation in practice size and deprivation levels in both groups. Six general practices will be recruited from the West of England region purposively, to include small and large practices and practices within different Primary Care Networks (PCNs)/clusters, which vary in deprivation as assessed by the Index of Multiple Deprivation. General practices will be approached by the West of England Clinical Research Network, which covers the Integrative Care Boards of: Bristol, North Somerset and South Gloucestershire; Bath and North East Somerset, Swindon and Wiltshire; and Gloucestershire. Using a proforma, baseline data will be collected from all six practice managers to describe their practice (Table 2).

**Table 2:** Data collection from six general practices recruited to the Vfrac feasibility study

**Feasibility study of Vfrac implementation within primary care**	**Outputs**
Outcome measures from intervention and control practices 1.1 Descriptives of each practice -Number of registered patients -Number of women ≥ 50 and ≥ 65 -Computer system -Name of PCN/cluster -Other demographic characteristics 1.2 Type of healthcare professional carrying out initial consultation for back pain 1.3 Number and date of consultations by older women with back pain 1.4 Resource use after initial consultation for back pain including -Consultations in primary and secondary care -Referrals including rheumatology, osteoporosis clinics, geriatrics, physiotherapy, pain clinic -Investigations including DXA, radiology -New medication prescriptions 1.5 New diagnoses since initial consultation for back pain Additional outcome measures from intervention practices only: 1.6 Proportion of consultations for back pain where Vfrac was used 1.7 Other consultations where Vfrac was used 1.8 Results of the Vfrac tool completed in primary care	1. Training requirements for healthcare staff 2. IT requirements and options for ideal integration within IT systems 3. Options for ideal integration within clinical pathways 4. Required length of follow-up for any future definitive trial to capture most (> 90–95%) patient journeys 5. Contribution to understanding of ‘usual care’ for patients who consult with back pain in the control practices where Vfrac is not used

**Nested qualitative assessment of acceptability**	**Outputs**
Outcome measures from participants recruited at intervention practices 2.1 Understanding of acceptability to healthcare professionals and patients 2.2 Identification of factors that impact on implementation, including barriers and facilitators to delivery	1. Results will form the basis of recommendations to modify Vfrac and improve delivery in any future trials

**Nested assessment of agreement between the use of Vfrac within general practices and self-completion at home**	**Outputs**
Outcome measures from participants recruited from intervention practices 3.1 Outputs of self-completion Vfrac 3.2 Satisfaction and ease of the use of self-completion questionnaire 3.3 Participants views of whether they preferred face-to-face assessment in primary care or self-completion at home 3.4 Assessment of agreement between outputs of the self-completed Vfrac and face-to-face Vfrac using Cohen’s kappa	1. Decision as to whether Vfrac can be carried out as a self-completion assessment or whether it needs to be delivered face-to-face for any future trial 2. Summary statistics for proportions of participants satisfied with process, interpersonal and technical attributes will be calculated 3. Recommendations in free-text fields of the satisfaction and ease of use questions will be used to modify the (future) tool if necessary

A clinical update on osteoporosis will be offered to all. Three practices will then be assigned to the intervention arm and three to the control by purposeful selection to ensure there are both large and small practices in both groups that have varying levels of deprivation. The control practices will use standard clinical processes for older women consulting with back pain. Those in the intervention arm will be trained in the use of Vfrac and will have it made accessible from their IT systems (through provision of a URL via pop-ups on typing relevant words such as back pain). The use of the Vfrac tool will be encouraged within their clinical pathways for management of older women (over 65 years of age) who consult with back pain. The use of the tool in a manner that is in accordance with each practice’s service delivery model will be facilitated by the research team in discussion with the clinical team. As the use of Vfrac poses only minimal/no risk, the use of a waiver of consent by individual patients for this cluster-level intervention is appropriate [15].

### Data collection

Data will be collected from the online Vfrac tool. When healthcare professionals enter data into the tool after clicking on the URL, inbuilt analytics will be used to record the Vfrac score, length of time taken and completeness of data collection. For each individual patient, once the data have been submitted, the Vfrac result (low or high risk of VFF) and recommendations will be available as a ‘copy and paste’, along with a unique Vfrac code (watermark). This will then be recorded in the individual patient’s medical records. Each practice will have their own URL allowing secure access to the online Vfrac tool, and also to allow analysis of differences in data collection across practices. No patient identifiable data will be recorded by the online Vfrac tool, the data stored will include the answers to each question for every entry per practice, the resulting Vfrac score, date submitted and unique Vfrac reference code generated per record. There will be no ability to link back any data to individual patients, only to practices. The Vfrac result and recommendation for each individual patient will have been copied and pasted into the medical records at the time of the consultation and will be captured during the electronic download from the general practices every three months using the watermark.

### Follow-up

Regular data will then be collected on consultations by older women with back pain from all six general practices every 3 months for 12 months. Data will be collected by electronic download to capture type of healthcare professional carrying out initial consultation for back pain, number and date of consultations by older women with back pain, resource use after initial consultation for back pain and new diagnoses since initial consultation for back pain (Appendix 1). In addition, from intervention general practices data will be collected on proportion of consultations for back pain where Vfrac was used, other consultations where Vfrac was used, and results of the Vfrac tool recorded in the medical notes (Appendix 2). Data collected will be anonymised at each general practice to remove patient identifiable details such as name, address and NHS number.

## WP 2: nested qualitative assessment of acceptability

A total of eight focus groups will be conducted, four with patients who were assessed using the Vfrac tool and four with healthcare professionals. Focus groups will aim to understand and assess the perceived acceptability of Vfrac and identify factors that impact on implementation, including barriers and facilitators to delivery. ‘Topic guides’ for patients and healthcare professionals will be used to guide discussions, with flexibility to pursue emerging ideas [16]. These will be informed by the Theoretical Framework of Acceptability [17], a framework that has been developed to guide the assessment of acceptability for providers and recipients, and implementation science theory [18], especially theories developed to explore the implementation of complex interventions. Similarities and differences between participants’ views will be explored. Initial focus groups will inform topic guide refinement.

Patients (female aged ≥ 65) who were assessed using the Vfrac tool during a consultation for back pain in intervention practices will be identified by healthcare professionals working within the practice. They will be identified by a unique code generated and embedded within their primary care records when Vfrac was used (with the prefix ‘VFRAC’). They will be approached by the direct clinical care team in their general practice with an information pack about the study. The information pack will contain an introductory letter, a participant information booklet, a consent form and a pre-paid envelope for return to the research team. Patients will be invited to contact the study team if they have any questions. Informed consent will be by self-completion of the consent form, after they have had time to read the patient information booklet and asked any questions. Those who reply by returning the completed consent form will be recruited. The consent form will be checked, initialled and dated by a Vfrac study team member. Patients who are unwilling to provide informed consent or who do not have the capacity to provide informed consent will be excluded. Purposive sampling will be used to take into account age, comorbidities and other relevant sociodemographic characteristics [19]. An estimated 4–6 patients will be included in each focus group, totalling 16–24 patients. The final sample size will be determined by data saturation [20]. If replies to the invitation in the study information packs are low (less than the required number of 16–24), a reminder letter will be sent 4–6 weeks after the original invitation.

Focus groups with patients will be conducted either in person on University of Bristol premises or remotely using video conferencing software. If face-to-face focus groups with patients are not feasible and individuals who have been recruited do not feel they have the necessary IT skills to participate in online group discussions, to ensure maximum inclusion they will also be given the option of taking part in one-to-one interviews to be conducted face-to-face or by telephone.

Healthcare professionals from intervention practices (WP1) who used Vfrac, or were involved in the implementation of Vfrac within IT systems or clinical pathways, will be approached via their research lead. Research leads will send an invitation email to potential participants with information about the study that will ask them to contact the research team if they are interested in taking part. Participants will include a range of healthcare professionals involved in the identification of VFFs in primary care including GPs, nurses, paramedics and first contact physiotherapists. Purposive sampling will be used to account for professional roles, years of experience and other relevant characteristics [21]. Written informed consent will be sought prior to data collection. After giving consent they will be asked to provide basic sociodemographic information (age, gender, ethnicity, professional grouping) and to indicate how they have been involved in setting up or using Vfrac. An estimated 4–6 participants will be included in each focus group, totalling 16–24 healthcare professionals. As above, the final sample size will be determined by data saturation [20]. If less than the required numbers of staff have replied (16–24), a reminder email will be sent 4–6 weeks after the original.

Focus groups with healthcare professionals will be carried out remotely using video conferencing software. If focus groups with healthcare professionals are not possible given their time constraints, provisions will be put in place for individual interviews that will either be conducted face-to-face, by telephone or using videoconferencing software to ensure maximum diversity and inclusion.

### Qualitative data analysis

All focus groups and interviews will be audio-recorded, transcribed and anonymised, then imported into NVivo qualitative analysis software. They will be transcribed through an approved company (The Transcription Company UK, https://www.thetranscription.co.uk/) with a confidentiality agreement in place between the company and the University of Bristol, using a standardised protocol used for all qualitative research at the University of Bristol. After the focus group or interview has been audio recorded on an encrypted device, it will be uploaded to the University of Bristol’s secure sever as soon as possible and then deleted from the audio recorder. The data file will then be uploaded to Transcription Company’s website using an encrypted file transfer service. It will then be transcribed in full and returned. Transcripts will then be anonymised by the research team.

Data from patients and healthcare professionals will be analysed as discrete datasets, using an inductive thematic approach to identify themes and subthemes in the responses [22]. Themes from both datasets will then be synthesised. To help understand the perceived acceptability of Vfrac, an abductive approach will be used (whereby the best supporting statements for emerging themes will be linked to each theme) with codes transposed into the ‘Theoretical Framework of Acceptability’ [23]. Further factors that impact implementation will also be transposed onto the normalisation process theory [18]. To illustrate this process, data will be displayed on charts using the framework approach to data organisation [24]. Factors identified by mapping codes onto the ‘Theoretical Framework of Acceptability’ and implementation science theory will be synthesised to form a taxonomy of barriers and facilitators to implementation. These will form the basis of recommendations to modify Vfrac and improve implementation and delivery in future trials and will be recorded in a live manual (manual 1B) which will be taken forward to the future definitive trial and eventual implementation, if appropriate.

## WP 3: nested assessment of agreement between health professional face-to-face completion of Vfrac and self-completion of Vfrac by patients

Older women (aged ≥ 65) who consulted with back pain and had Vfrac used during their clinical consultation within one of the three intervention practices will be recruited to take part in this work package. They will be identified by the unique code generated and embedded within their GP records at the time the result of Vfrac was recorded.

Eligible patients will be approached by the direct clinical care team in their general practice with an information pack about the study. The information pack will contain an introductory letter, a participant information booklet, a consent form, a paper version of the Vfrac tool they can self-complete at home, plus a pre-paid envelope for return to the research team. Those who reply by returning the completed consent form and self-completed paper version of Vfrac will be recruited. The consent form will be checked, initialled and dated by a Vfrac study team member. If no reply is received, a reminder letter will be sent 4–6 weeks after the original mailing. Patients who are unwilling to provide informed consent or who do not have the capacity to provide informed consent will be excluded.

The questionnaire for self-completion at home will include the published Vfrac questions [14] plus questions on satisfaction and ease of use at home compared to during the consultation for back pain at their general practice. The additional questions on satisfaction and ease of use were based on the framework on Quality in Healthcare developed by Huycke and All [25] to cover process, interpersonal and technical attributes and relevant questions from the validated questionnaire on remote consultations by Mekhjian et al. [26]. The method for self-measurement of the wall-to-tragus distance was based on a published method [27] and work with our experienced in-house musculoskeletal Patient and Public Involvement (PPI) group to produce easy to use instructions for measurement at home.

Radiology data from medical records will be accessed by EC to assess the presence or absence of VFFs. This is necessary, as there is a concern that people with vertebral fractures may find it difficult to measure their wall-to-tragus distance due to difficulty raising their arms above head height [28].

After receiving the completed consent forms and questionnaire, the study team will provide practices with patient identifiable data (names, date of birth and address) of patients who have been recruited to this nested assessment of agreement. Electronic primary care records will then be accessed by the practice team to identify the unique Vfrac reference number for each patient recruited to this nested study and this will be shared with the study team to assess the agreement between face-to-face completion by a healthcare professional and self-completion by the patient.

### Quantitative data analysis

Outputs (high risk versus low risk of VVF) of the self-completion Vfrac questionnaire will be compared with those from the face-to-face Vfrac completed at the general practice during WP1, and agreement assessed using Cohen’s kappa. Responses to the satisfaction and ease of use questions will if necessary be used to modify the tool for future use.

## Sample size

### WP1: Vfrac implementation within primary care

A sample size of six general practices has been chosen because discussions with primary care have identified three potential operational characteristics that may impact on uptake, ease of use and acceptability of Vfrac. These are IT system (EMIS Web and SystmOne), size (small versus large) and within different PCNs/clusters. The recruitment of six practices will enable us to observe and explore the various aspects of feasibility within and across these various characteristics.

### WP2: nested qualitative assessment of acceptability

An estimated 4–6 participants will be included in eight focus groups, totalling 16–24 patients and 16–24 healthcare professionals. As noted above, the final sample sizes will be determined by data saturation [29].

### WP3: nested assessment of agreement between the use of Vfrac within general practices and self-completion at home

Based on assessment of agreement using Cohen’s kappa, and assuming approximately 30% of Vfrac outputs will be classified as high risk, for a sample size of 60 (20 from each intervention practice), the margins of error from 95% confidence intervals around estimates of kappa in the range 0.8–0.6 (the definition of substantial agreement) would be from 0.16 to 0.22. This level of precision is deemed sufficient for these purposes.

## Outcome measures and outputs

### Study status

Recruitment for the study began in September 2022 and the study is anticipated to be complete by May 2024.

## Discussion

This is the first study to assess a new clinical decision tool to identify which older women presenting to primary care with back pain are at risk of having an existing VFF and should be referred for a spinal radiograph. The FRAX clinical tool is available to identify an individual’s future risk of VFF, but it differs from Vfrac in that it does not give any information on risk of existing VFFs, instead providing a probability of future fracture [30]. The Study of Osteoporotic Fractures (SOF) has identified regression models to predict those with VFFs [31] and concluded that it was better to perform radiographs in all white women aged ≥ 70 with low bone mass. This is unlikely to be a useful strategy, as assessment of bone mass is not carried out at a population level, and spinal radiographs impart a dose of ionising radiation equivalent to an entire year of background radiation [32]. Simple clinical tools to guide osteoporosis management within primary care have proven to be effective, as many (such as FRAX) are in common use in clinical practice today. Vfrac provides a targeted method of identifying those with existing VFF that could run alongside FRAX. The Vfrac decision tool has an area under the curve (AUC) of 0.802 (95% CI, 0.764–0.840) and it identified 93% of those with more than one fracture and two-thirds of those with one fracture [14].

Feasibility work is required before a large study can be designed to investigate the extent to which Vfrac improves the treatment of older people with osteoporosis. Specifically, this feasibility work will help assess the real-world clinical delivery and implementation of Vfrac and address key uncertainties around trial parameters including sample size required for the cluster design (based on numbers of women who attend their GP with backpain as well as an indication of the potential degree of clustering) and length of follow-up required (based on the time it takes from assessment to final management). It will also generate important information to understand the acceptability of Vfrac to healthcare professionals and patients and identify barriers, facilitators and contextual factors that impact on its implementation. In turn, this will help develop a series of recommendations to optimise the Vfrac tool and its delivery. Results from the second nested study will determine whether Vfrac can be used remotely, that is, as a self-completion tool or as part of a remote consultation. In addition, this feasibility study will assess whether appropriate data on resource use can be collected in a future clinical trial to provide a definitive estimate of the cost-effectiveness of Vfrac.

One of the limitations of this feasibility study is that the use of the Vfrac tool will only be evaluated in older women and the data collected would be confined to older women. Further work is being conducted as part of a separate study to determine whether the Vfrac tool can be used to identify vertebral fractures in older men with a similar success rate to older women. We will assess the level of agreement in Vfrac outputs obtained from face-to-face completion with a research nurse and self-completion by men. Semi-structured interviews will also be undertaken to determine if the Vfrac tool is acceptable to men. Another limitation of this study is the recruitment of only six general practices which will offer only limited data that does not account for UK-wide variability in consultation, management and healthcare use for back pain. Therefore, we will collect and analyse anonymized national data acquired from the Clinical Practice Research Datalink to investigate UK-wide variability in consultation, management and healthcare utilisation for back pain in older adults. This will help understand what ‘usual care’ entails for older people with back pain and help plan future testing of the Vfrac tool.

A future parallel group cluster randomized trial will determine the clinical- and cost-effectiveness of Vfrac from the NHS perspective, compared with the standard care of older women with back pain. The stop/go criteria for a future cluster randomized controlled trial informed by this feasibility study will rely on a realistic required sample size (of up to 40 clusters), a realistic required length of follow-up (no greater than 15 months), and evidence that the Vfrac tool is acceptable to healthcare professionals and patients.

In conclusion, this study is being conducted in preparation for a future definitive, cluster randomized controlled trial to fully evaluate the use of the Vfrac decision tool in improving the detection of VFFs in older women presenting to primary care with back pain. Outputs of this study will allow accurate evidence-led development, planning and funding of the future definitive evaluation of the clinical- and cost-effectiveness of Vfrac. If successful, national implementation of Vfrac is likely to identify more of the currently undiagnosed older women with VFFs. By appropriate interventions with medications that reduce their future fracture risk by 30–70%, implementation of Vfrac is likely to have a major benefit in reducing fracture risk within the older population. It would certainly change clinical practice for older women with back pain.

## Data Availability

The data sets generated from this study will be available in the University of Bristol Research Data Repository. Data will be available 6 months following publication of the feasibility study findings. Access to the data will be restricted to ensure that data are only made available to bona fide researchers for ethically approved research projects, on the understanding that confidentiality will be maintained and after a data access agreement has been signed by an institutional signatory.

## Appendix 1. Vertebral fracture clinical decision tool for older women with back pain (Vfrac)—a feasibility study

### Follow-up data collection form: control general practices

(A). Practice name
(B). Date of download
(C). Data required for
  - All women > 50
  - With consultations for back pain since start of study
(D). List of data to download in an anonymised format*

**Table:**

Anonymised ID
Date of birth
Date of initial consultation for back pain *{now called initiating event}*
Details of initiating event
Professional grouping of healthcare worker carrying out initiating event, e.g. doctor, nurse, and first contact physio
List of other consultations within general practice since initiating event
Medications at time of initiating event
Changes in medications since initiating event—with date of change
Referrals to secondary care since initiating event—with date of referral
Radiology referrals since initiating event—with date of referral
DXA referrals since initiating event—with date of referral
Diagnoses since initiating event—with date of diagnosis

*Patient level data to be anonymised by general practices before returning to research team.

## Appendix 2. Vertebral fracture clinical decision tool for older women with back pain (Vfrac)—a feasibility study

### Follow-up data collection form: intervention general practices

(A). Practice name
(B). Date of download
(C). Data required for
  - All women > 50
  - With consultations for back pain since start of study
(D). List of data to download in an anonymised format*

**Table:**

Anonymised ID
Date of birth
Date of initial consultation for back pain *{now called initiating event}*
Details of initiating event
Professional grouping of healthcare worker carrying out initiating event, e.g. doctor, nurse, and first contact physio
Vfrac unique code (watermark)
Output of Vfrac (recommend X-ray; no X-ray recommended; value)
List of other consultations within general practice since initiating event
Medications at time of initiating event
Changes in medications since initiating event—with date of change
Referrals to secondary care since initiating event—with date of referral
Radiology referrals since initiating event—with date of referral
DXA referrals since initiating event—with date of referral
Diagnoses since initiating event—with date of diagnosis

*Patient level data to be anonymised by general practices before returning to research team.
